# CC chemokines and receptors in osteoarthritis: new insights and potential targets

**DOI:** 10.1186/s13075-023-03096-6

**Published:** 2023-07-03

**Authors:** Yuchen Zhang, Di Liu, Djandan Tadum Arthur Vithran, Bosomtwe Richmond Kwabena, Wenfeng Xiao, Yusheng Li

**Affiliations:** 1 Department of Orthopedics, Xiangya Hospital, Central South University, Changsha, 410008 Hunan China; 2 Xiangya School of Medicine, Central South University, Changsha, 410013 Hunan China; 3 National Clinical Research Center for Geriatric Disorders, Xiangya Hospital, Central South University, Changsha, 410008 Hunan China

**Keywords:** CC chemokine, CC chemokine receptor, Osteoarthritis, Chemokine, Inflammation

## Abstract

Osteoarthritis (OA) is a prevalent degenerative disease accompanied by the activation of innate and adaptive immune systems-associated inflammatory responses. Due to the local inflammation, the expression of various cytokines was altered in affected joints, including CC motif chemokine ligands (CCLs) and their receptors (CCRs). As essential members of chemokines, CCLs and CCRs played an important role in the pathogenesis and treatment of OA. The bindings between CCLs and CCRs on the chondrocyte membrane promoted chondrocyte apoptosis and the release of multiple matrix-degrading enzymes, which resulted in cartilage degradation. In addition, CCLs and CCRs had chemoattractant functions to attract various immune cells to osteoarthritic joints, further leading to the aggravation of local inflammation. Furthermore, in the nerve endings of joints, CCLs and CCRs, along with several cellular factors, contributed to pain hypersensitivity by releasing neurotransmitters in the spinal cord. Given this family’s diverse and complex functions, targeting the functional network of CCLs and CCRs is a promising strategy for the prognosis and treatment of OA in the future.

## Background

In the early twentieth century, osteoarthritis (OA) was merely considered a “wear-and-tear” mechanically driven disease with aging. However, accumulating evidence has indicated that OA is a systemic musculoskeletal disease that involves the activation of innate and adaptive immune systems accompanied by inflammation [1]. OA is typically characterized by chronic pain, joint instability, stiffness, and joint deformities, all of which severely affect the patients’ quality of life [2]. However, the pathophysiological mechanisms for OA have not been fully understood, and disease-modifying drugs have remained a challenge for OA treatment. For advanced OA patients, total knee arthroplasty (TKA) was an inevitable choice, which causes a heavy socio-economic burden [3]. Therefore, it is crucial to further investigate the pathogenesis and therapeutic targets of OA.

Local inflammatory processes are crucial in the initiation and progression of OA, and chemokines play a vital role in the homing and mobilization of stem cells and progenitor cells, regulating their migration during tissue homeostasis [4]. Based on the precise configuration of the two cysteines closest to the N terminus, chemokines can be classified into different subfamilies [5]. CC motif chemokine ligands (CCLs) are one of the essential members of chemokines whose cysteines are directly juxtaposed. The specific receptors of CCLs are CC motif chemokine ligands receptors (CCRs), which belong to G protein-coupled receptor family [6]. Currently, 28 CCLs and 10 CCRs have been described [7].

**Fig. 1:**
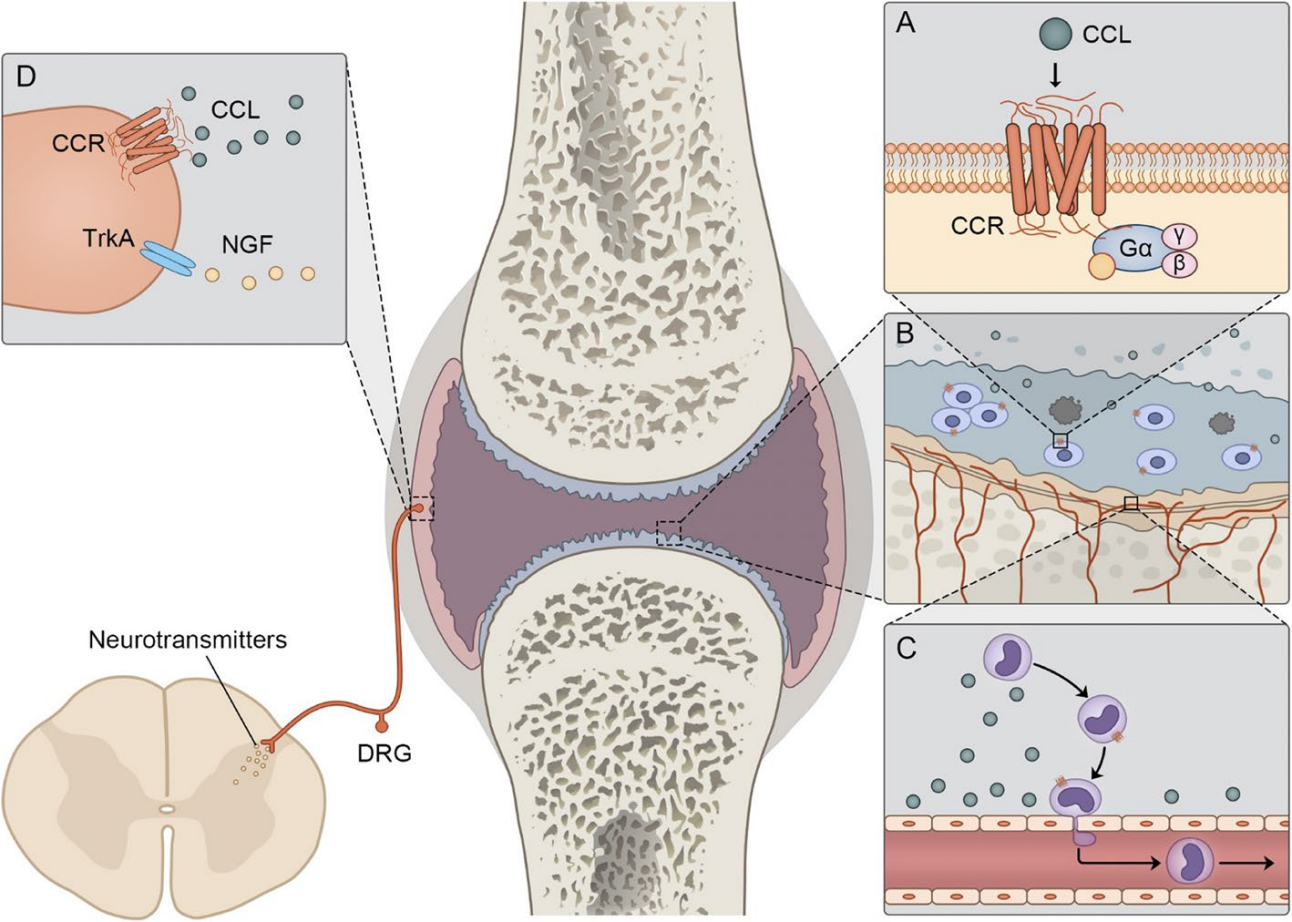
Pathogenesis in OA related to CCLs and CCRs. CCLs and CCRs participate in the pathogenesis and development of OA through multiple mechanisms. CCL binds to specific CCR, activating downstream biochemical processes via the G-protein-coupled pathway (**A**), which promotes chondrocyte apoptosis (**B**) and upregulates the expression of multiple matrix-degrading enzymes in the cartilage. The binding of CCL and CCR also induces the infiltration of various immune cells into the damaged joint, leading to local inflammation (**C**). At the nerve endings in the joint, the binding of CCL and CCR, as well as other related cell factors such as NGF and its receptor TrkA (**D**), leads to the release of neurotransmitters in the spinal cord, causing pain hypersensitivity. CCL, CC motif chemokine ligand; CCR, CC motif chemokine ligands receptor; OA, osteoarthritis; NGF, nerve growth factor; TrkA, tropomyosin receptor kinase A; DRG, dorsal root ganglia

The interaction between CCLs and CCRs is pivotal in both the pathogenesis and treatment of OA. As chemokines, CCLs attracted immune cells into osteoarthritic joints in the early stage and promoted the formation of an inflammatory microenvironment [8]. In addition, CCLs promoted the expression levels of matrix metalloproteinases (MMPs) and a disintegrin and metalloproteinase with thrombospondin activity (ADAMTSs), which contributed to the cartilage degradation during OA progression [9, 10]. Moreover, CCLs were involved in chondrocyte apoptosis as well as osteoblast and osteoclast differentiation in OA joints [8, 11, 12] and influenced the nervous system leading to pain responses, which may be mediated by CCLs binding to CCRs on neural cells [10] (Fig. 1). Furthermore, targeting CCLs and CCRs may become a novel strategy to alleviate clinical manifestations and delay OA progression.

In this review, we summarized the role of CCLs and CCRs in the occurrence and development of OA and shed light on the latent diagnostic and therapeutic value of CCLs and CCRs in OA.

### Structure and function of CCLs and CCRs

The CC family is one of the largest among chemokines, and CCRs are a subfamily of the G protein-coupled receptor (GPCR) [13]. The interaction between CCLs and CCRs can recruit and activate diverse immune cells, such as monocytes/macrophages, neutrophils, T cells, natural killer (NK) cells, fibroblasts, and endothelial cells, which participate in the pathogenesis of OA [14].

When CCLs bind to their corresponding CCRs, they induce the exchange of guanosine diphosphate/guanosine triphosphate (GDP/GTP) on the Gα subunit [15]. This results in the activation of downstream signaling pathways, including but not limited to the adenylate cyclase (AC)/cyclic adenosine monophosphate (cAMP)/protein kinase A (PKA) pathway. Additionally, G proteins are also involved in the activation of the phospholipase C (PLC), phospholipase A2 (PLA2), and phosphodiesterase (PDE) signaling pathways [16]. Indeed, the signaling pathways activated by CCLs and CCRs play a critical role in the pathogenesis of OA, including the initiation and progression of the disease.

### CCL and CCR expression in OA

The expression of many chemokines is altered in OA joints, including both inflammatory and homeostatic types. Inflammatory chemokines are associated with inflammation, while homeostatic chemokines are constitutively expressed in specific tissues and cells and have diverse functions, such as inducing stem cell migration [17]. There are also some “dual-function” chemokines [7] (Table 1). For the CCL family, inflammatory chemokines include CCL2, CCL3, CCL5, CCL7, CCL8, CCL11, CCL13, CCL14, CCL15, CCL24, CCL26, CCL27, and CCL28; homeostatic chemokines include CCL18, CCL19, and CCL21; and dual-function chemokines include CCL1, CCL17, CCL20, CCL22, and CCL25 [18]. Multiple studies have shown that chemokines were significantly upregulated in rheumatoid arthritis (RA) [19–22]. Elevated levels of CCLs and CCRs were also detected in the synovium and synovial fluid in OA patients [23]. Intriguingly, although the expression of multiple CCLs was elevated in OA compared to normal donors, there was also a partial decrease in chemokine expression [24].

**Table 1:** The conventional classification of CCLs and their affinity receptors

Chemokine (other name)	Receptor
**Inflammatory**	
CCL2 (MCP-1)	CCR2
CCL3 (MIP-1α)	CCR1, CCR5
CCL5 (RANTES)	CCR1, CCR3, CCR5
CCL7 (MCP-3)	CCR1, CCR2, CCR3
CCL8 (MCP-2)	CCR1, CCR2, CCR5
CCL11 (Eotaxin-1)	CCR3, CCR5
CCL13 (MCP-4)	CCR2, CCR3
CCL14 (HCC-1)	CCR1, CCR3, CCR5
CCL15 (HCC-2)	CCR1, CCR3
CCL24 (Eotaxin-2)	CCR3
CCL26 (Eotaxin-3)	CCR3
CCL27 (CTACK)	CCR10
CCL28 (MEC)	CCR3, CCR10
**Homeostatic**	
CCL18 (DC-DK1)	PITPNM3
CCL19 (ELC)	CCR7
CCL21 (SLC)	CCR7
**Dual-Function**	
CCL1 (I-309)	CCR8
CCL17 (TARC)	CCR4
CCL20 (LARC)	CCR6
CCL22 (MDC)	CCR4
CCL25 (TECK)	CCR9

Normal chondrocytes were found to express certain CCLs (such as CCL2, CCL3, CCL4, CCL5, CCL8, and CCL20) and CCRs (such as CCR1, CCR2, CCR3, and CCR5) [25–27]. In a study, Borzi et al. [27] demonstrated that CCLs and CCRs could stimulate catabolic activities in chondrocytes, indicating a potential role in cartilage homeostasis. Under pro-inflammatory conditions, the expression of CCL2, CCL3, CCL4, CCL5, CCL8, and CCL20 was significantly elevated in chondrocytes [26]. In OA chondrocytes, the expression of CCL2, CCL3, CCL4, CCL18, CCL20, CCL22, CCR1, CCR2, CCR3, CCR5, and CCR6 was upregulated [20, 25–29]. Interestingly, CCL5 induced the expression of its receptor, CCR5, suggesting an autocrine/paracrine pathway of the chemokine in cartilage [30]. OA chondrocytes produced CCL11 in response to stimulation with interleukin-1β (IL-1β) and tumor necrosis factor-α (TNF-α) [31]. CCL22 was also detectable in damaged cartilage and induced chondrocyte apoptosis [32].

Several investigations have demonstrated increased expression of CCLs and CCRs in the synovial tissues of individuals with OA [20, 33–38]. The synovium contains synovial macrophages and synovial fibroblasts (SFs). Compared to normal SFs, it was found that human OA synovial fibroblasts (OASFs) expressed higher levels of CCL5 and CCR5 [39]. The levels of CCL2 were observed to be significantly elevated in the synovial fluid of OA patients compared to healthy controls [40]. Chemerin and its receptor were expressed in human SFs, which enhanced the expression of toll-like receptor-4 (TLR-4) and the release of CCL2 through a post-transcriptional mechanism while moderately attenuating SFs proliferation [41]. OASFs secreted CCL2 in response to stimulation by cartilage lesions and hepatocyte growth factor (HGF) [23, 42]. Connective tissue growth factor (CTGF) promoted the production of IL-6, IL-8, CCL2, and CCL20 in SFs in the presence of IL-1β [43]. OASFs also constitutively expressed CCR2, CCR5, CXCR3, and CXCR4, but not CCR3 and CCR6 [44]. CCL17 was expressed in synovial macrophages and regulated by interferon regulatory factor 4 (IRF4) and granulocyte–macrophage colony-stimulating factor (GM-CSF) in the GM-CSF-dependent collagenase-induced OA model [45]. The protein content of synovial fluids from OA patients was higher than that from normal donors with CCL2, CCL3, and CCL4 increased in the synovial fluid. These CCLs are primarily produced by the inflamed synovium and damaged cartilage in OA [46].

Moreover, the expression level of CCL11 was upregulated in inflamed osteoblasts both in situ and in vitro, and further analysis revealed that CCL11 was co-localized with its high-affinity receptor CCR3 [47]. Lisignoli et al. [19] have reported high expression levels of CCR6 in osteoblasts from patients with OA and RA. Additionally, CCL9 and its homologous receptor CCR1 were upregulated in osteoclasts [48]. These findings suggested that CCLs and CCRs may significantly impact the regulation of bone resorption in OA.

Furthermore, the serum levels of CCLs and CCRs were altered in OA patients. Elevated levels of CCL2, CCL3, and CCL11 have been detected in the peripheral blood of OA patients to some extent, implying their potential utility as biomarkers for the diagnosis and prognosis of OA [31, 49, 50]. Serum CCL2 levels may be useful in deciphering some mechanisms of joint destruction in OA and identifying individuals with a higher probability of structural changes in the knee joint [51]. Notably, a subgroup analysis revealed that the serum CCL2 level was significantly higher in Chinese, Dutch, and Brazilian OA patients [50].

Differentially expressed CCLs and CCRs in various joint tissues contributed to variable biological effects, some of which were closely associated with the pathological features and clinical symptoms of OA. Hence, CCLs and CCRs showed tremendous potential as diagnostic markers and therapeutic targets.

### CCLs and CCRs in biochemical cascades

The combination of CCLs and CCRs led to phenotypic changes through the biochemical cascades of G protein-coupled receptors [52], and this process, by negative feedback, formed a complex regulatory network. The nuclear factor-kappa B (NF-κB) signaling pathway regulated several CC chemokine family members, including CCL2, CCL4, CCL18, and CCL20 [53–55]. NF-κB is a family of transcription factors. Articular chondrocytes have been shown to activate NF-κB through the expression of CCLs. This activation led to a significantly promoted expression of MMPs and ADAMTSs, resulting in cartilage lesions and chondrocyte apoptosis in OA [56, 57]. Yang et al. [58] have also demonstrated that microRNA-495 can bind to CCL4, resulting in the downregulation of post-transcriptional CCL4 expression and subsequent inhibition of the NF-κB signaling pathway, leading to a decrease in chondrocyte apoptosis induced by CCL4-NF-κB axis.

CCL2 plays a potentially causal role in the occurrence and development of OA. Appleton et al. [59] have found that CCL2/CCR2 induced cartilage degradation through the TGF-α/CCL2 axis, and blockade of CCL2 reduced the loss of proteoglycans and digestion of type II collagen structure, as well as downregulation of MMP-3 in OA. Vascular cellular adhesion molecule-1 (VCAM-1) was known to participate in leukocyte adhesion and transmigration to the interstitium during inflammation [60]. In human OASFs, CCL2 increased the expression of VCAM-1 via the CCR2, PKCδ, p38MAPK, c-Jun, and AP-1 signal pathways. This induction of VCAM-1 expression by CCL2 promoted the adhesion of monocytes to human OA synovial fibroblasts [40]. The addition of CCL2 or CCL5 can induce MMP-3, inhibit the synthesis of proteoglycans, and enhance the proteoglycan release of chondrocytes through the interaction of their receptors CCR2 and CCR5 [30]. CCL2/CCR2 and CCL5/CCR5 also increased collagenase and gelatinase activities in SFs [44]. The chemokine system may contribute to cartilage degradation in OA in an autocrine or paracrine manner, and the absence of CCL2 or CCR2 strongly suppressed particular inflammatory response genes in articular joints, including arginase 1, prostaglandin synthase 2, nitric oxide synthase 2, inhibin A, IL6, MMP3, and tissue inhibitor of metalloproteinases 1 [61].

CCL3 overexpression can cause a decrease in the expression of cartilage-specific genes and increase MMP3, MMP13, ADAMTS-4, and ADAMTS-5 expression, indicating its potential role in regulating degradation in primary human chondrocytes [28]. CCL5 and CCR5 interact through PKC/c-Src/c-Jun and AP-1 signaling pathways to increase IL-6 production in human SFs and accelerate the OA progression [39]. In normal conditions, elevated CCL5 enhanced the expression of MMP-1, inducible nitric oxide synthase (iNOS), and IL-6 expression in chondrocytes and increased glycosaminoglycan (GAG) release in cartilage [62].

CCL11, also called eotaxin-1, has been shown to play a crucial role in the catabolic processes of chondrocytes by enhancing the expression of CCR3 and CCR5 on chondrocyte surfaces and inducing the expression of MMP-3 and MMP-13 in osteoarthritic joints [31]. Borzi et al. [27] have reported that CCL2, CCL4, CCL5, and other C-X-C chemokines induced MMP-3 release from chondrocytes. CCL11 on SFs promoted the MMP-9 release by activating the CCR3 signaling pathway, and its expression was also induced by IL-1 and TNF in OASFs [63], indicating that inflammatory factors could contribute to the upregulation of CCLs in OA.

In CCL17-deficient mice, the expression of MMPs was decreased, and joint destruction was alleviated [45]. Moreover, the interplay between CCR7 and its ligands CCL19 and CCL21 can stimulate SF migration, and stimulation of SFs with CCL19 increased VEGF secretion, promoting synovial angiogenesis and aggravating synovial inflammation [64]. In addition, the upregulation of CCL21 has been shown to induce ECM loss and play a crucial role in cartilage damage through the crosstalk between the meniscus and synovium [65].

CCL20, which activated the Akt phosphorylation signaling pathway, was associated with increased b-N-acetylhexosaminidase release in CCL20-stimulated OA osteoblasts [19]. Its mRNA expression was low in normal chondrocytes but increased after stimulation by proinflammatory cytokines. In the presence of CCL20, the release of MMP-1, MMP-13, PGE2, GAG fragment, and IL-6 in cartilage significantly increased, while collagen type II mRNA expression was inhibited, and collagen type X mRNA expression was stimulated, indicating that CCL20 was a positive factor for OA progression [29, 66].

CCL22, which was involved in chondrocyte inflammation and cartilage degradation in OA with CCR4, is downregulated and attenuates downstream CCR4-related signaling pathways, inhibiting cartilage inflammation in OA [67]. Knockdown of CCL22 has been shown to suppress the secretion of pro-inflammatory cytokines in OA murines, including CCL2, TNF-a, IL-1β, IL-6, p-p65, and COX2, while overexpression of CCR4 increased their expression, indicating the promoting effect of CCL22 on OA inflammation. CCL25 has also been shown to have a pro-inflammatory effect by causing a reduction of GAG and collagen type II expression and inducing the expression of MMP1, MMP3, early growth response protein 1 (EGR1), and superoxide dismutase 2 (SOD2) in porcine chondrocytes cultured in an OA model, indicating its potential involvement in cartilage degradation [68].

Based on the abovementioned evidence, the elevated expression of multiple CCLs in OA leads to further disease progression through biochemical cascades. One important mechanism is the increased expression of MMPs and ADAMTSs, key enzymes in cartilage matrix degradation.

### CCLs and CCRs in chondrocyte apoptosis and subchondral bone remodeling

Chondrocytes are the only resident cells in articular cartilage that are indispensable for maintaining extracellular matrix. In the development of OA, compromised chondrocyte function and survival can lead to cartilage lesions, resulting in irreversible damage to articular cartilage, with chronic pain and loss of mobility [69, 70]. Under normal conditions, increased CCL2 promotes chondrocyte apoptosis and inhibits proliferation [8]. Previous studies have shown that CCR5 − / − mice developed less cartilage degeneration, indicating the potentially protective role of CCR5-ablation in cartilage homeostasis [71]. Similarly, CCR7 − / − mice had reduced cartilage erosion and osteophytes after destabilization of the medial meniscus (DMM) surgery [72]. CCL22 had a functional role in apoptotic chondrocytes [32], and its expression correlated with TNF-α activation, which played a role in chondrocyte apoptosis [73, 74]. Therefore, CCL22 may mediate TNF-α-induced chondrocyte apoptosis.

Subchondral bone remodeling is another important morphological change observed in OA joints and is associated with a dynamic balance between osteoblasts and osteoclasts [75]. CCLs and CCRs promoted the differentiation and maturation of osteoblasts and osteoclasts, playing an essential role in subchondral bone changes and osteophyte formation [11, 12]. CCL3 promoted osteoclast formation through CCR1 or CCR5 [76].

Promoting of chondrocyte apoptosis and subchondral bone remodeling by CCLs and CCRs may contribute to OA progression. Despite numerous observed phenotypes, the specific molecular mechanisms of how CCLs and CCRs lead to articular cartilage and bone damage require further investigation.

### CCLs and CCRs in cellular chemotaxis

CCLs exhibit potent chemotactic effects on various immune cells, including neutrophils, eosinophils, basophils, monocytes, mast cells, dendritic cells, NK cells, T lymphocytes, and B lymphocytes [77], thereby contributing to the complex immune microenvironment in OA. These chemotactic effects can promote OA tissue changes and clinical symptoms.

After a joint injury, CCL2 expression levels were significantly upregulated, which recruited CCR2 + monocytes, leading to local inflammation in damaged articular tissues [78]. CCR2 + macrophages have been found near the articular cartilage surface and may directly contribute to OA cartilage degradation and tissue damage [23]. CCL2 and CCR2 were upregulated in the dorsal root ganglia (DRG) of knee nerves in OA mice models, and the CCL2/CCR2 axis was critical for recruiting macrophages to sites of injury. In contrast, no macrophage infiltration was observed in CCR2 − mice [79]. Similarly, the CCL3/CCR1 axis attracted circulating monocytes to the inflamed synovium in OA, while blocking either the CCL3/CCR1 or the CCL2/CCR2 axis was shown to reduce synovial hyperplasia and macrophage infiltration in OA knees [46].

However, while CCR2 was the receptor for CCL2, there was a lack of direct correlation between CCL2 and CCR2. According to a study by Jablonski et al. [80], macrophages showed differences in number, location, and phenotype after full-thickness cartilage defect injuries in mice lacking CCL2, CCR2, or both. They found that CCR2 inhibited cartilage regeneration after injury, whereas CCL2 acted as a protective role and was required for the differentiation or functional integrity of mesenchymal stem cells during cartilage regeneration. Moreover, CCL2 enhances the expression of MMP3 and MMP13 in chondrocytes by attracting macrophages and other inflammatory cells [8].

Chemokines present in synovial fluid may play a role in the recruitment of multifunctional progenitor cells from the subchondral bone marrow to the site of the microfracture defects. CCL25 in synovial fluid can recruit human subchondral mesenchymal progenitors, while CCL22 and CCL27 inhibited cell migration, and CCL2 and CCL24 had no effect [24]. CCLs and CCRs contributed to synovitis in OA, which was marked by hyperplasia of the synovial lining, fibrosis of the sublining, and vascularization of the stromal tissue [81]. CCR4 was mainly expressed by CD4 + T cells and endothelial cells in synovium, and CCL22 was increased locally in inflamed synovium, promoting synovitis [33]. To sum up, the chemotactic effects of CCLs in synovial fluid and synovium contributed to OA progression through osteophyte production and synovitis.

Osteophyte production was also closely associated with osteoblasts and osteoclasts. CCL11 increased pre-osteoclast migration in vitro, and CCR3 receptor expression colocalized with CCL11 in osteoclasts at the bone surface [47]. This suggested that the CCL11/CCR3 pathway was important for the migrating pre-osteoclasts to the bone surface, promoting the destruction of native bone. A separate investigation demonstrated that the CCR7/Rho signaling pathway promoted osteoclast migration and resorption activity in response to CCL19 and CCL21, leading to subchondral bone remodeling [21]. Inflammation upregulated CCR7 expression in osteoclasts, which promoted their recruitment to the site of bone remodeling. The recruitment of osteoblasts and osteoclasts by CCLs and CCRs accelerated subchondral bone remodeling and the formation of osteophytes.

Mesenchymal stem cells (MSCs) are a type of multipotent stem cells that are crucial in repairing cartilage [82]. CCL2 can recruit MSCs to the injury site while triggering changes in the MSCs’ transcriptome, leading to a block in the chondrogenic program and limiting their escape [70]. CCL25 was a potent inducer of MSCs, recruiting them to OA cartilage in a porcine model [68]. CCL21 has been shown to significantly increase the migration of CCR7-expressing MSCs from the bone marrow of young rabbits and promote cartilage repair [83].

The chemotactic effects of CCLs on various immune cells and functional stem cells promoted the development of OA inflammation, and osteophyte generation due to abnormal bone remodeling. However, some CCLs recruited MSCs to promote cartilage repair, delaying OA progression. Thus, targeted interventions against CCLs hold great potential for OA treatment.

### CCLs and CCRs in pain-related behavior

Pain is an important protective mechanism in the body. OA patients have lower pain thresholds that probably be associated with altered central sensitization, but the exact mechanism is not fully understood [84]. Certain CCLs and CCRs are involved in pain-related behaviors in OA. The most well-understood is the relationship between CCL2/CCR2 and OA pain-related behaviors. A study conducted on OA patients found a significant increase in the levels of CCL2 in synovial fluid, and a positive correlation between CCL2 levels and pain [85]. In the DMM model of OA in mice, both CCL2 and CCR2 were upregulated in DRG of the knee joint and pain-related behaviors (e.g., decreased locomotion) were observed compared to the control group, which may be associated with macrophage infiltration into DRG. However, macrophage infiltration was not observed in CCR2-null mice, and these mice did not develop movement-provoked pain behaviors, suggesting a key role for the CCL2/CCR2 pathway in osteoarthritis pain [79]. A similar conclusion was reached in the experiment of Ishihara et al. [86]. Additionally, Miller et al. [10] indicated that blocking ADAMTS5 after DMM could reduce the CCL2 level in DRG neurons and temporarily reverse mechanical allodynia. These findings suggested that CCL2/CCR2 axis may have specific involvement in pain-related mechanisms in OA. CCL2-CCR2 signaling pathway at the joint contributed to knee hyperalgesia in OA, partially mediated by direct stimulation of CCR2 expressed by sensory afferents within the affected joints [87].

CCL17 was also associated with OA-related pain via the GM-CSF-CCL17 pathway, and CCL17 had a chemotactic-independent function in this process [88]. GM-CSF induced the transcription factor IRF4, which then mediated the pro-inflammatory and algesic functions by regulating the production of CCL17 [89]. The GM-CSF-CCL17 pathway led to the activation of downstream mediators, including nerve growth factor (NGF), neuropeptides CGRP, and substance P, which were crucial for developing inflammatory pain [90, 91]. Inhibitors of CCL17 can reduce the peripheral pain effect in CiOA model rats [45]. Furthermore, in a rat model of joint injury, CCL22 was positively correlated with pain-related behaviors [32].

Most of the mechanisms mentioned above differ from chemokine’s classical role. However, their classic chemokine role may indirectly cause pain by promoting inflammation.

### Targeting CCL and CCR networks in OA

Targeting the functional network of CCLs and CCRs to treat OA is promising. The measurement of CCLs and CCRs levels may provide valuable predictive information regarding the occurrence, development, and recurrence of OA. For instance, increased expression of CCL2 has been observed in OA patients compared to healthy controls, suggesting its potential involvement in the progression of OA [92]. Circulating CCL2 levels have been suggested as a possible biomarker for the diagnosis of OA [50, 51]. Moreover, the presence of CCL2 gene polymorphisms has been linked to an increased susceptibility to OA and could serve as potential markers for early diagnosis of the condition [93]. Guo et al. [49] found that CCL2 and CCL3 levels were considerably higher in the bone and joint tissues of OA rats, as well as in the peripheral blood of OA patients. They also showed that the levels of CCL2 and CCL3 in the peripheral blood can be good diagnosis indicators and have predictive value for the curative effect and prognosis of OA recurrence. Similarly, the levels of CCL13 in serum and synovial fluid were positively correlated with the radiographic severity of OA, indicating their potential as a biomarker for OA progression [94].

In addition to their predictive value, blocking the CCLs and CCRs functional network related to OA has broad prospects for treatment. CCR2 blockade has been shown to significantly delay joint damage progression in OA, with different structural outcomes depending on the duration of the blockade [95]. This suggested that regulating the polarization of macrophages through CCR2 at an early stage may be crucial for joint recovery after injury. Therefore, precise modulation of inflammation at the appropriate site and timing may be essential for effective intervention [96].

Furthermore, the HOTTIP/mir-455-3p/CCL3 regulatory network played a key role in OA pathogenesis, indicating that blocking CCL3 expression via long non-coding RNA (lncRNA) HOTTIP was a latent target for OA treatment [28]. Another study has found that hsa_circ_0134111, a circular RNA (circRNA), was overexpressed in the cartilage of patients with OA and played a role in the progression of OA by interacting with miR-224-5p, which alleviated the inhibitory effect of miR-224-5p on CCL1 [97]. These findings suggested that certain lncRNA and circRNA also have the potential as a target for curing OA.

In another study, CCL25-supplemented hyaluronic acid (HA) injection significantly alleviated OA scores by reducing cartilage proteoglycan loss and knee cartilage destruction [98]. Furthermore, CCL19 and CCL21 stimulated the migration of osteoclast precursor cells and the differentiation of osteoclasts via the Rho-ROCK signaling pathway. Targeted blockade of Rho-ROCK signaling or CCL19 and CCL21 may therefore ameliorate subchondral bone remodeling in OA [21].

Given the role of CCLs and CCRs in OA, they have great potential as predictive indicators and therapeutic targets, which may provide novel insights for diagnosing and treating OA.

## Conclusion

Although OA affects the quality of life of the aging population, the specific mechanisms remain unclear, and disease-modifying treatments are still limited. On the surface of the cellular membrane, CCL binds to a specific CCR with high affinity, activating downstream biochemical cascades via the G-protein-coupled pathway. This activation promoted chondrocyte apoptosis and upregulated the expression of multiple matrix-degrading enzymes in cartilage. Moreover, the binding of CCLs and CCRs induced the infiltration of various immune cells into the damaged joint, leading to local inflammation. At the nerve endings in the knee joint, CCLs and CCRs, along with several cell factors, cause the release of neurotransmitters in the spinal cord, resulting in pain hypersensitivity (Table 2). Therefore, clarifying the multifaceted biofunctions of CCLs and CCRs family in osteoarthritic cartilage may contribute to the early-term diagnosis and alleviation of OA.

**Table 2:** Summary of biofunctions of CCLs in OA

Chemokine	Model type	Function summary
**Biochemical cascades**		
CCL2	In vitro, mice	Increase VCAM-1 expression Induce MMP-3 secretion Inhibit proteoglycan synthesis Increase collagenase and gelatinase activity
CCL3	In vitro	Decrease the expression of cartilage-specific genes Increase expression of MMP3, MMP13, ADAMTS-4, and ADAMTS-5
CCL5	In vitro	Increase expression of MMP-1, iNOS, and IL-6 Increase GAG release
CCL11	In vitro	Increase CCR3 and CCR5 expression Increase MMP-3 and MMP-13 expression
CCL19	In vitro	Increase VEGF secretion
CCL20	In vitro	Increase b-N-acetylhexosaminidase release Increase release of MMP-1, MMP-13, PGE2, GAG fragment, and IL-6 Inhibit collagen type II mRNA expression Stimulate collagen type X mRNA expression
CCL21	In vitro	Induce ECM loss
CCL22	In vitro	Increase secretion of CCL2, TNF-a, IL-1β, IL-6, p-p65, and COX2
CCL25	In vitro	Decrease expression of GAG and collagen type II Induce expression of MMP1, MMP3, EGR1, and SOD2
**Chondrocyte apoptosis and subchondral bone remodeling**		
CCL2	In vitro, mice	Promote chondrocyte apoptosis while impeding cellular proliferation
CCL3	In vitro, mice	Promote osteoclast formation
CCL22	In vitro	Mediate TNF-α-induced chondrocyte apoptosis
**Cellular chemotaxis**		
CCL2	In vitro, in vivo, mice	Recruit monocytes to the OA joint Recruit macrophages to injured DRG Recruit MSCs to the OA joint Induce transcriptome changes in MSCs
CCL3	In vitro, in vivo	Attracted circulating monocytes to the inflamed synovium
CCL11	In vitro, mice	Increase pre-osteoclast migration in vitro
CCL19	In vitro	Promote migration and resorption activity of osteoclasts
CCL21	In vitro	Promote migration and resorption activity of osteoclasts Recruit MSCs to OA cartilage in rabbit model
CCL22	In vitro	Inhibit mesenchymal progenitors migration
CCL25	In vitro	Recruit subchondral mesenchymal progenitors Recruit MSCs to OA cartilage
CCL27	In vitro	Inhibit mesenchymal progenitors migration
**Pain-related behavior**		
CCL2	In vivo, mice	Contribute to knee hyperalgesia Modulate sensory afferents in the affected DRG
CCL17	In vitro, mice, rat	Induce inflammatory pain mediators
CCL22	Rat	Associated with pain-related behaviors

## Data Availability

Not applicable.

## Abbreviations

OA: Osteoarthritis
CCL: CC motif chemokine ligand
CCR: CC motif chemokine ligand receptor
TKA: Total knee arthroplasty
MMP: Matrix metalloproteinase
ADAMTS: A disintegrin and metalloproteinase with thrombospondin activity
NGF: Nerve growth factor
TrkA: Tropomyosin receptor kinase A
DRG: Dorsal root ganglia
GPCR: G protein-coupled receptor
NK cells: Natural killer cells
RA: Rheumatoid arthritis
IL-1β: Interleukin-1β
TNF-α: Tumor necrosis factor-α
SF: Synovial fibroblast
IRF4: Interferon regulatory factor 4
GM-CSF: Granulocyte-macrophage colony-stimulating factor
NF-κB: Nuclear factor-kappa B
VCAM-1: Vascular cellular adhesion molecule-1
GAG: Glycosaminoglycan
DMM: Destabilization of the medial meniscus
MSC: Mesenchymal stem cell
